# Ultrasound in Renal Cancer Screening: A Review of the Literature

**DOI:** 10.7759/cureus.83790

**Published:** 2025-05-09

**Authors:** Lennox Ksido, Sally Zhou, Julia Glatman, Eve Frangopoulos, Sawania Christolin, Lulu Wei, Nicholas Karanikolas, Jeffrey P Weiss

**Affiliations:** 1 Department of Urology, State University of New York Downstate Medical Center, Brooklyn, USA; 2 Department of Urology, Vanderbilt University Medical Center, Nashville, USA

**Keywords:** early detection, literature review, renal cancer, screening, ultrasound

## Abstract

Kidney cancer is a prevalent cancer that is often asymptomatic at early stages and is commonly incidentally diagnosed using cross-sectional imaging. This review aims to provide a comprehensive review describing the utility of ultrasound, a low-cost, safe, and widely accessible tool, as a potential method for renal cancer screening. We conducted a comprehensive literature search using PubMed and Embase and identified 177 articles pertaining to ultrasonography screening of renal cell carcinoma. Fifteen articles met the following inclusion criteria: studies that used renal ultrasonography, studies that used abdominal ultrasound, studies that found renal cancer incidentally, and studies written in English. Key findings from these studies highlighted ultrasound as an effective tool for detecting asymptomatic renal cancers and identified a need to optimize screening guidelines to minimize costs and lower overdiagnosis. Further, they showed that a screening program can particularly benefit from including patients with known risk factors including older age, male gender, smoking history, and immunosuppression. We propose integrating kidney screening into existing abdominal ultrasound screening guidelines to potentially enhance feasibility and cost-effectiveness.

## Introduction and background

Renal cell carcinoma (RCC) is a common urological malignancy in the United States with a 76% five-year survival rate and is the seventh most prevalent neoplasm in the developed world [1,2]. Its incidence in the United States has more than doubled in the last 50 years due to the increasing use of imaging that enables incidental detection of small, asymptomatic tumors [1]. While surgeons can often cure early-stage kidney cancer, advanced disease has a poor prognosis. The five-year relative survival rate for stage I disease is 93% as compared to 72.5% for stage II/III and only 12% for stage IV [2].

Early detection may save lives, yet routine screening for RCC is not currently recommended due to concerns about cost-effectiveness and overdiagnosis. Ultrasound (US) has emerged as a potential screening tool due to its affordability, widespread availability, and lack of radiation or contrast exposure. Compared to computerized tomography (CT) and magnetic resonance imaging (MRI), ultrasound offers a more affordable, safer, and non-invasive alternative, making it an attractive option for screening. Given the similarities between RCC risk factors and those for abdominal aortic aneurysms (for which there are established screening guidelines), integrating renal ultrasound into existing preventive health initiatives could be a practical and efficient approach.

This manuscript evaluates the available evidence on ultrasound as a screening tool for RCC, assessing its feasibility, diagnostic accuracy, and potential role in screening programs. Further, we identify knowledge gaps and propose a standardized methodology for future research examining optimized RCC screening strategies.

Methods

Literature Search Strategy

**Figure 1:**
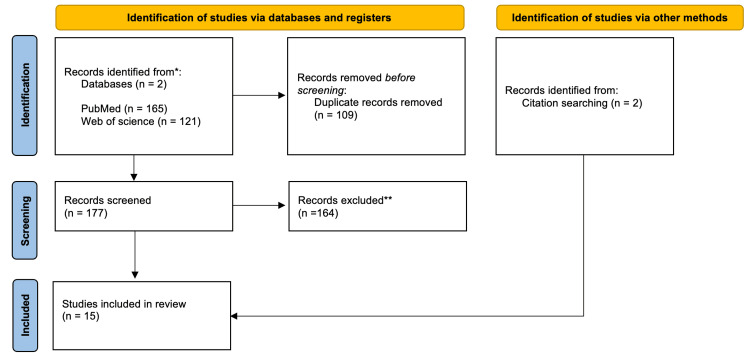
Literature search strategy: Preferred Reporting Items for Systematic Reviews and Meta-Analysis (PRISMA) flow diagram

We conducted a comprehensive literature search (Figure 1) to identify relevant articles from January 1982 to December 2023 following the guidelines of the Preferred Reporting Items for Systematic Reviews and Meta-Analysis (PRISMA) [3]. We searched PubMed and Embase databases for English-language articles using the following terms: ("renal cancer" OR "renal cell carcinoma" OR "incidental detection") AND ("ultrasound" OR "ultrasonography") AND "screening"). The title and abstract of each article were analyzed, and relevant articles were identified for further review. Three reviewers then read the full text, collaborating to determine which articles met the predefined criteria for inclusion. A consensus method was used to resolve disagreements. To identify any additional sources, we examined the reference list of the articles. The selected studies were then categorized based on type of study, population screened, and risk factors present.

Selection Criteria

Through our search, 177 articles pertaining to ultrasonography screening of RCC were identified. Our team then selected clinical trials that specifically addressed screening of asymptomatic individuals (i.e., no evidence of disease including pain, palpable mass, changes in weight, change in urination). We included articles that described incidental findings of renal cancer, studies that used renal ultrasonography, and studies that used abdominal ultrasound. We excluded studies conducted on symptomatic patients and studies utilizing CT as the primary imaging modality. We also excluded 18 studies not written in English. Fifteen studies were ultimately selected for analysis; nine studies investigating asymptomatic populations and six studies investigating renal transplant patients.

## Review

Nine studies used ultrasound to screen asymptomatic patients, often as part of routine health assessments (Table 1) [4-12]. While most studies examined the kidneys alone, a subset of three studies extended their screening by utilizing abdominal ultrasound [7,8-10]. Those studies detected significantly more abnormalities compared to studies examining the kidneys alone. We also identified six studies that screened individuals who had either undergone kidney transplants or were on the transplant waiting list (Table 2) [13-18]. 

**Table 1:** Screening of the general population ‡Abdominal ultrasound † weighted average *Retrospective study (included both asymptomatic patients and patients with lower urinary tract symptoms). AML: Angiomyolipoma, RCC: Renal Cell Carcinoma

Author (Country)	No. of Subjects	Age	Gender (% male)	Suspected vs. Diagnosed RCC	Avg. Size of Tumor (cm)	Tumors under 3cm	Tumors over 3cm	No. of Pts with Metastasis	No. of alternative findings	Most common finding
Tosaka et al., 1990^‡ ^[8] (Japan)	41364	N/a	N/a	355:19	5	N/a	N/a	0	336	Cysts (82)
Fujii et al., 1995 [4] (Japan)	17941	Median 53y	72%	45:20	4.5	N/a	N/a	N/a	35	AML (24)
Spouge et al., 1996^‡ ^[10] (Canada)	1000	Mean 46.2y	91%	4:4	N/a	N/a	N/a	0	415	Fatty liver (142)
Mihara et al., 1999 [6] (Japan)	219640	N/a	N/a	439:192	4.7^†^	65 < 2.5cm	107 > 2.5 cm	0	185	Benign tumors (84)
Tsuboi et al., 2000 [9] (Japan)	60604	N/a	67%	97:13	N/a	6	7	N/a	28	AML (24)
Mizuma et al., 2002^‡ ^[7] (Japan)	16024	Mean 47y	58%	24:5	4.2	0	5	N/a	106	Hydronephrosis (56)
Filipas et al., 2003 [11] (Germany)	9959	Mean 61y	49%	13:9	6	4	7	2	276	Kidney stones (214)
Malaeb et al., 2004 [5] (USA)	6678	Mean 66.2y	97%	22:15	5.8	1	14	1	817	Cysts (627)
Haliloglu et al., 2010* [12] (Turkey)	18203	Mean 55y	64%	81:36	4.19	5	31	1	38	AML (35)

**Table 2:** Screening in kidney transplant patients ‡ Screened ESRD patients before transplant *Retrospective studies. RCC: Renal Cell Carcinoma

Author (Country)	No. of Subjects	Mean Age	Gender (% male)	Suspected vs. Diagnosed RCC	Avg. Size of Tumor	Tumor under vs. over 3cm	No. of Pts with Metastasis
Gulanikare et al., 1998^‡ ^[15] (USA)	206	N/a	61.7%	8:8	2cm	N/a	0
Schwarz et al., 2007 [17] (Germany)	561	51.7y	56.9%	11:8	N/a	N/a	N/a
Filocamo et al., 2009* [14] (Italy)	604	46.7y	N/a	10:10	2.1cm	10:0	0
Bennett et al., 2017 [13] (USA)	135	M68, F48	58.3%	4:4	4.7cm	2:1	0
Kiss et al., 2022* [16] (Hungary)	1687	52.3y	58%	26:18	2.6cm	N/a	1
Yohanna et al., 2023* [18] (USA)	1998	N/a	N/a	N/a:20	N/a	N/a	3

The primary objective of the general population studies was to assess the incidence of RCC detection and determine the validity of screening. Several articles also commented on the five-year survival of diagnosed patients. Demographically, the study populations were predominantly male, however, the gender distribution varied significantly. Filipas et al. and Malaeb et al. reported male populations of 49% and 97%, respectively [5,11]. Such variability may contribute to discrepancies in incidence calculations as RCC is more prevalent in men. The reported incidence of RCC ranged from 0.046% in Tosaka et al.’s study to 0.4% in Spouge et al.’s study, likely reflecting differences in study populations [8,10]. The average size of tumors ranged from 4.19 cm to 6 cm [11,12]. Secondary findings such as cysts or kidney stones were also commonly reported on, providing additional benefit for patient care.

Six articles described RCC screening programs in asymptomatic renal transplant patients [13-18]. These articles reported higher incidences of renal cancer in renal transplant patients than in the general population. Of note, the tumors were, on average, smaller than in the general population. A possible explanation could be the short duration between renal transplant and the onset of the screening program (typically one to three years after transplant), allowing the tumors to be caught earlier.

Key findings

Our review uncovered three critical areas important in evaluating the practicality of ultrasound in screening for RCC.

Effectiveness of US for the Early Detection of RCC

Using a cancer registry to track their participants, the study by Mizuma et al. was able to determine the sensitivity and specificity of abdominal screening sonography in detecting abdominal cancers, determining it to be 78.6% and 95.3%, respectively. Notably, out of the 11 cancers detected by screening, six patients survived after resection, five of whom had RCC. The increase in survival among renal cancer patients further underscores the potential of focusing sonographic screening on kidneys [7]. Filipas et al. also attempted to define the sensitivity and specificity of US for kidney cancer detection through a multi-phase screening program in which participants were screened twice, one year apart. Similarly to Mizuma et al., the sensitivity and specificity of US imaging for kidney cancer were found to be 82% and 98%, respectively [11].

Ultrasound proved to be effective at detecting small renal tumors. The study by Mihara et al. involving 219,640 individuals undergoing abdominal US screening found 192 cases of RCC. Of the 172 patients that underwent resection, 37.8% of tumors were less than 2.5 cm in size [6]. Mihara et al. also described specific US features commonly seen in small RCCs. Such findings included the presence of a marginal hypoechoic zone, an anechoic component, and a protrusion of the tumor from the surface of the kidney. Educating sonographers on these cancer markers, may increase detection rates and decrease false negatives. 

Targeting Asymptomatic, High-Risk Populations

Asymptomatic patients with underlying risk factors for RCC are the optimal population to screen. Mihara et al. found that elevated blood urea nitrogen (BUN) and creatinine levels were seen in only 13.8% and 5.8% of diagnosed patients, respectively [6]. This finding demonstrates that renal cancer is often asymptomatic and shows normal results on kidney function analysis. Tosaka et al. found a threefold higher likelihood of RCC diagnosis in symptomatic patients compared to asymptomatic ones [8]. The latter group, however, had significantly smaller masses that were lower in clinical stage. Strikingly, the asymptomatic group had a significantly higher five-year post-nephrectomy survival rate (94.7% as compared to 60.9%, p<.01). No significant difference in tumor grade between the two groups was observed. Similarly, Mihara et al.'s study, which screened asymptomatic patients, found that the survival rate after nephrectomy was 97.4% at five years and 94.6% at 10 years. These findings support the utility of proactive screening in asymptomatic populations.

The studies conducted by Malaeb et al. and Spouge et al. were those with the greatest incidence of RCC detection: 0.22% and 0.4% respectively. Malaeb et al. examined a population of older, predominantly male veterans, a group likely to have more RCC risk factors, reinforcing the value of targeting higher-risk groups [5]. Spouge et al., who examined asymptomatic executives, found the greatest incidence of RCC at 0.4% [10]. These studies had the two highest percentages of men as research participants, 97% and 91% respectively, further reinforcing the concept of using risk factors in determining the population to screen.

Optimizing Screening Protocols

Enhancing screening protocols may improve cost-effectiveness and minimize some of the undesirable consequences such as overdiagnosis of benign conditions and false positives. The studies by Fuji and Mihara revealed that some cases of angiomyolipomas are not distinguishable from RCC, necessitating monitoring [4,6]. Filipas et al. suggested that repeating US for suspicious findings could reduce the rate of false positives [11]. Furthermore, Tsuboi et al. proposed monitoring smaller tumors via US and CT imaging instead of further workup [9]. Annual monitoring until confirmation of diagnosis can be a potential method to avoid over-treatment and is supported by high five-year survival rates for tumors of small size [19].

Limitations and challenges

A limitation of our review is the potential underestimation of RCC incidence due to participant dropout or refusal to continue care after identification of a lesion. We also observed differences in the level of expertise among those performing the ultrasound exams. Operator experience ranged from a single physician completing the imaging procedure to a varied group of imaging specialists. Tosaka et al. used a physician specializing in imaging diagnosis, whereas Mizuma et al. had a team of medical technologists conduct the ultrasound, differences that can potentially skew comparative results [7,8]. Further, retrospective designs of several studies restricted the scope of data collection. Lastly the exclusion of non-English studies introduces a language bias.

We thus conducted a risk of bias assessment of the included studies in Table 3. QUADAS-2 was selected as the risk of bias tool because most included studies evaluated screening tests for RCC using imaging and then further confirmed the results. We found that most studies appropriately selected patient populations and used valid reference standards. The poor consistency of ultrasound interpretation and the variability in patient follow-up, however, contributes to uncertainty in the results. These findings underscore the need for better-designed prospective studies.

**Table 3:** Risk of Bias Summary for Included Studies Using QUADAS-2 Criteria Low - low risk of bias, Unclear - unable to determine bias, High - high risk of bias.

Study	Patient Selection	Index Test	Reference Standard	Flow & Timing
Tosaka et al., 1990 [8]	Low	Unclear	High	High
Fujii et al., 1995 [4]	Low	Unclear	High	High
Spouge et al., 1996 [10]	Low	Unclear	High	High
Mihara et al., 1999 [6]	Low	Unclear	Low	Low
Tsuboi et al., 2000 [9]	Low	Unclear	High	High
Mizuma et al., 2002 [7]	Low	Unclear	Low	Low
Filipas et al., 2003 [11]	Low	Unclear	Low	Low
Malaeb et al., 2004 [5]	Low	Unclear	Low	Low
Haliloglu et al., 2010 [12]	Low	Unclear	Low	Low
Gulanikar et al., 1998 [15]	Low	Unclear	Low	Low
Schwarz et al., 2007 [17]	High	Unclear	Low	High
Filocamo et al., 2009 [14]	High	Unclear	Low	High
Bennett et al., 2017 [13]	High	Unclear	Low	High
Kiss et al., 2022 [16]	High	Unclear	Low	High
Yohannan et al., 2023 [18]	High	Unclear	Low	High

Establishing a standardized methodology

Reported outcomes varied amongst all nine studies despite describing similar endpoints, highlighting the need for consistent data collection in future studies. Commonly reported metrics were population demographics, the number of participants that necessitated further review after US, and the number of confirmed RCCs diagnosed. Reports on the size of the tumors at diagnosis and the prevalence of metastasis, however, differed markedly. Diagnostic confirmation methods also varied, with most studies diagnosing based on pathology and a minority diagnosing with CT, percutaneous fine needle aspiration, and angiography. Further, our review revealed variations in recorded data points. Some studies counted the number of tumors under/over 3 cm, while one used 2.5 cm as the cutoff. These inconsistencies hinder analysis, reinforcing the need for uniform data collection in future studies. In Table 4, we propose standard data collection points essential for the robust analysis of screening. 

**Table 4:** Recommended parameters for RCC screening by US studies RCC: Renal Cell Carcinoma

Category	Utility in RCC Screening
Number of subjects	Provides a baseline for statistical power and representation in the study.
Average age of subjects	Older age is a risk factor for RCC; understanding age distribution can help correlate risk.
Gender of subjects	RCC is more prevalent in males thus, this category is crucial for gender-specific screening strategies.
Presence of risk factors	Recording risk factors such as smoking, hypertension, and obesity aids in determining the population to be screened.
# of subjects with suspicious findings	The number of patients that undergo further examination informs the assessment of feasibility and cost.
# of subjects diagnosed by histology	Confirms diagnosis post-screening, allows for assessment of the specificity of ultrasound.
Average size of tumor found	Tumor size is critical for staging and treatment strategies as well as determining risk of metastasis.
Tumors smaller vs. greater then 3cm	Tumors under 3 cm are unlikely to have metastasized leading to better outcomes after surgery.
Number of patients with metastasis	Provides insights into the ability to discover tumors before they spread and treatment becomes less effective.
5-year survival	A key indicator of long-term treatment success and screening program effectiveness.
10-year survival	Further establishes the longevity of treatment outcomes post screening.
# of patients with metastasis at 5/10 years	Critical for understanding the progression of the disease and long-term effectiveness of early detection.

Benefits of early detection of renal cancer

RCC is challenging to diagnose; the classic triad of hematuria, flank pain, and palpable abdominal mass is rarely seen in clinical practice [2]. Instead, symptoms tend to be vague and non-specific, with many patients remaining asymptomatic until advanced stages. Currently, nearly half of RCCs are discovered incidentally, which can be detrimental because there is a steep drop in median survival and five-year survival rates in locally invasive and metastatic stages of RCC [2]. Implementing a screening tool increases the probability of detecting RCC at a smaller tumor size, which is directly linked to the risk of metastasis [20]. Metastatic disease risk is negligible in patients with tumor size less than 3 cm, however, the mean size upon detection of RCC is 5 cm. In a clinical cohort completed by Kunkle and his team, they found that with each 1 cm increase in tumor size beyond 3 cm, there was a 22% increased risk of metastasis [20]. The risk of metastasis is doubled at a 3.5 cm increase in size. In the studies we examined, ultrasound was found to be effective at detecting renal tumors smaller than 3 cm. Early treatment of renal cancer can potentially reduce the healthcare and economic burdens associated with advanced stages of the disease, which typically require costly chemotherapy, more frequent emergency room visits, and palliative surgeries.

Risk factors and high-risk populations

Understanding the risk factors of RCC will help guide the population to screen. Renal cancer incidence increases steadily with age, with a peak incidence of 64 years old in the USA [21]. Men are more commonly affected by RCC, with a male-to-female ratio of approximately 2:1. Cigarette smoking is one of the most well-established modifiable risk factors for RCC. Smokers have been found to have a significantly increased risk of developing RCC and tend to have more aggressive disease [22]. Obesity also has a positive association with the risk of developing this malignancy [23]. It is important to highlight the increased risk of developing RCC in patients who have undergone solid organ transplantation, particularly kidney transplantation. Prolonged immunosuppression may impair immune surveillance mechanisms predisposing to post-transplant RCC [16]. Kidney transplant patients are often screened for kidney cancer, setting a precedent for expanding the indications for screening to other risk factors.

Discussion

This review highlights the potential of ultrasound as a screening tool for RCC in high-risk populations. The implementation of such a program would lead to the early detection of renal cancer, improving long-term survival rates and potentially lowering the burden of chronic disease on the health system. Renal cancer, specifically, is ideal for screening due to its slow growth and potential for curative treatment if discovered early. Existing small-scale screening programs for renal transplant patients can be used as a model for future programs.

The studies examined in this paper consist of 15 trials in which the feasibility and efficacy of screening were reviewed. They demonstrated that ultrasound is effective at detecting renal cancer, including small tumors. However, the studies encountered a low incidence of detection. We recommend screening patients with known risk factors rather than the general population to increase incidence rates. Some risk factors for RCC include male gender, advanced age, a history of smoking, obesity, and immunosuppression. Expanding renal ultrasound to include abdominal ultrasound may improve cost-effectiveness by increasing the number of diseases screened for little extra cost. Abdominal ultrasound screening is already recommended for men aged 65 to 75 years who have a history of smoking in order to detect abdominal aortic aneurysms [24]. As these screening guidelines include three common risk factors for RCC, wouldn't it make sense to screen for both simultaneously? To further improve the feasibility and cost-effectiveness of a screening program, active surveillance for small renal masses is recommended and can be used to decrease overdiagnosis. Rose et al. found that imaging at three months, six months, and every six months for up to three years, followed by annual surveillance, is effective at reducing overtreatment and conserves healthcare resources [25].

When considering a screening program for kidney cancer, drawing parallels with colon cancer, a disease that has a well-established screening program, may be insightful. Like renal cancer, colon cancer is slow-growing, detectable by screening (colonoscopy), and highly treatable at early stages. While colon cancer is more prevalent in the USA compared to renal cancer (104,610 new cases of colon cancer in 2020 [26] compared to about 81,610 new cases of kidney cancer in 2024 [27]), ultrasound for RCC is cheaper and less invasive than colonoscopy.

Ultrasound does have some limitations. Abdominal ultrasounds can be challenging in obese patients due to the impairment of image quality of the abdominal anatomy [28]. Further, while ultrasound can identify a mass, it does not provide information on the histological type or grade of the tumor, necessitating additional imaging or biopsy for a definitive diagnosis and classification. Further work-up is needed to differentiate between benign and malignant lesions, adding to costs and subjecting the patient to more invasive work-up procedures. Ultrasound is also not effective at detecting distant metastases compared to CT or MRI. Lastly, ultrasound is user-dependent, with successful detection varying based on the sonographer’s expertise.

## Conclusions

Ultrasound has demonstrated strong potential as a cost-effective, accessible, and non-invasive screening tool for kidney cancer, particularly in high-risk populations. Our review of the existing literature found that ultrasound can effectively detect small renal tumors, which are associated with improved survival rates. Despite its promise, ultrasound-based RCC screening faces several challenges including a lack of a standardized screening program, operator dependence, and uncertainty regarding cost-effectiveness. Further research is needed to fully realize the benefits of ultrasound and to investigate a potential integration into the established screening for abdominal aortic aneurysms. Additionally, a randomized controlled trial should be conducted to determine if screening high-risk individuals would reduce RCC-related mortality.
